# Irreducible Lisfranc Injury Caused by Tibialis Anterior Tendon Entrapment: Surgical Strategy and Literature Review

**DOI:** 10.7759/cureus.109895

**Published:** 2026-05-29

**Authors:** Satoshi Muto, Hideyuki Ota, Tadayori Handa, Hiroaki Kumagai, Toshihisa Kojima

**Affiliations:** 1 Department of Orthopedic Surgery, Nagoya Medical Center, Nagoya, JPN; 2 Department of Orthopedic and Hand Surgery, Nagoya Ekisaikai Hospital, Nagoya, JPN

**Keywords:** irreducible dislocation, lisfranc fracture dislocations, midfoot injury, open reduction internal fixation, tibialis anterior tendon

## Abstract

Lisfranc injuries are difficult to treat, particularly when reduction is blocked by soft-tissue interposition. Tibialis anterior tendon entrapment is a rare cause of irreducible Lisfranc dislocation, with only nine cases reported since 1950. We report the tenth case in a 65-year-old woman who sustained a Lisfranc fracture-dislocation after a trampoline accident. Emergency treatment consisted of closed reduction and temporary percutaneous fixation, followed by staged open reduction and internal fixation after soft-tissue swelling subsided. At definitive surgery, the tibialis anterior tendon was found entrapped between the medial and middle cuneiforms, preventing reduction. After release of the tendon, anatomic alignment was restored, and stable fixation was achieved. Although the tendon remained entrapped for 14 days, the patient had a favorable recovery, with progressive improvement in range of motion and an American Orthopedic Foot and Ankle Society midfoot score of 90 at six months. This case emphasizes the need to consider tendon interposition in irreducible Lisfranc injuries and suggests that staged surgical management may yield favorable outcomes in this uncommon setting.

## Introduction

Lisfranc injuries are among the most challenging foot injuries to reduce and stabilize. Lisfranc injuries involve the tarsometatarsal joint complex, which connects the metatarsals to the medial, middle, and lateral cuneiforms and the cuboid. This joint complex plays an important role in maintaining midfoot stability and the transverse arch of the foot. The tibialis anterior tendon runs along the medial aspect of the foot and inserts mainly onto the medial cuneiform and the base of the first metatarsal, contributing to ankle dorsiflexion and inversion. Improper reduction can lead to joint instability and post-traumatic arthritis, which can significantly impair functional outcomes; therefore, prompt and appropriate treatment is essential [1,2]. Management of Lisfranc injuries includes closed or percutaneous fixation, open reduction and internal fixation with screws or dorsal bridge plates, and primary arthrodesis in selected cases [3-7]. Regardless of the fixation method, restoration of anatomic alignment is critical because residual incongruity is associated with poor functional outcomes and post-traumatic arthritis [1,2]. However, in some cases, specific factors complicate reduction and make management particularly difficult. One such rare phenomenon is the interposition of the tibialis anterior tendon at the site of injury. In Lisfranc injuries, when the tibialis anterior tendon becomes entrapped between the medial and middle cuneiforms, it can act as a mechanical block and prevent successful closed reduction. To date, only a limited number of cases have been reported [8-11]. Notable among these are the case reports by Karaindros et al. and Kim et al., both of which identified tibialis anterior tendon interposition as a cause of irreducible Lisfranc injury [10,11]. However, the incidence, injury mechanism, and preoperative diagnostic value of this condition remain unclear. Therefore, failure to achieve or maintain closed reduction should raise suspicion of soft-tissue interposition, including tibial anterior tendon entrapment. This report aims to describe a rare case of irreducible Lisfranc fracture-dislocation caused by tibialis anterior tendon interposition, successfully treated with staged surgery, and to discuss its diagnostic and therapeutic implications in the context of the existing literature.

## Case presentation

**Figure 1:**
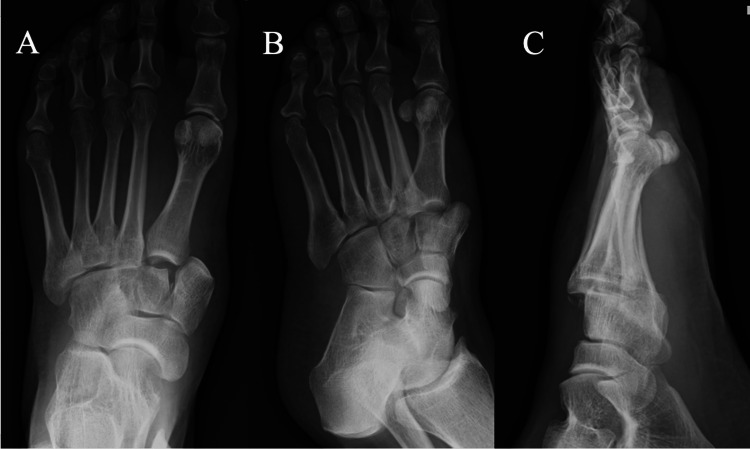
Preoperative radiographs of the left foot showing a Lisfranc fracture dislocation (A) Anteroposterior view. (B) Medial oblique view. (C) Lateral view. The images show the dislocation of the tarsometatarsal joint complex, widening between the medial and middle cuneiforms, and the key joints involved. These findings suggest marked midfoot instability and may raise suspicion of soft-tissue interposition in an irreducible Lisfranc injury.

A 65-year-old woman was referred to our hospital after injuring her left foot during a trampoline landing accident. On initial examination, she presented with swelling, pain, and deformity of the left midfoot and was unable to bear weight. Her medical and medication history was unremarkable. Radiographs showed a Lisfranc fracture-dislocation with malalignment of the medial column and widening between the medial and middle cuneiforms, and emergency surgery was planned (Figure 1).

**Figure 2:**
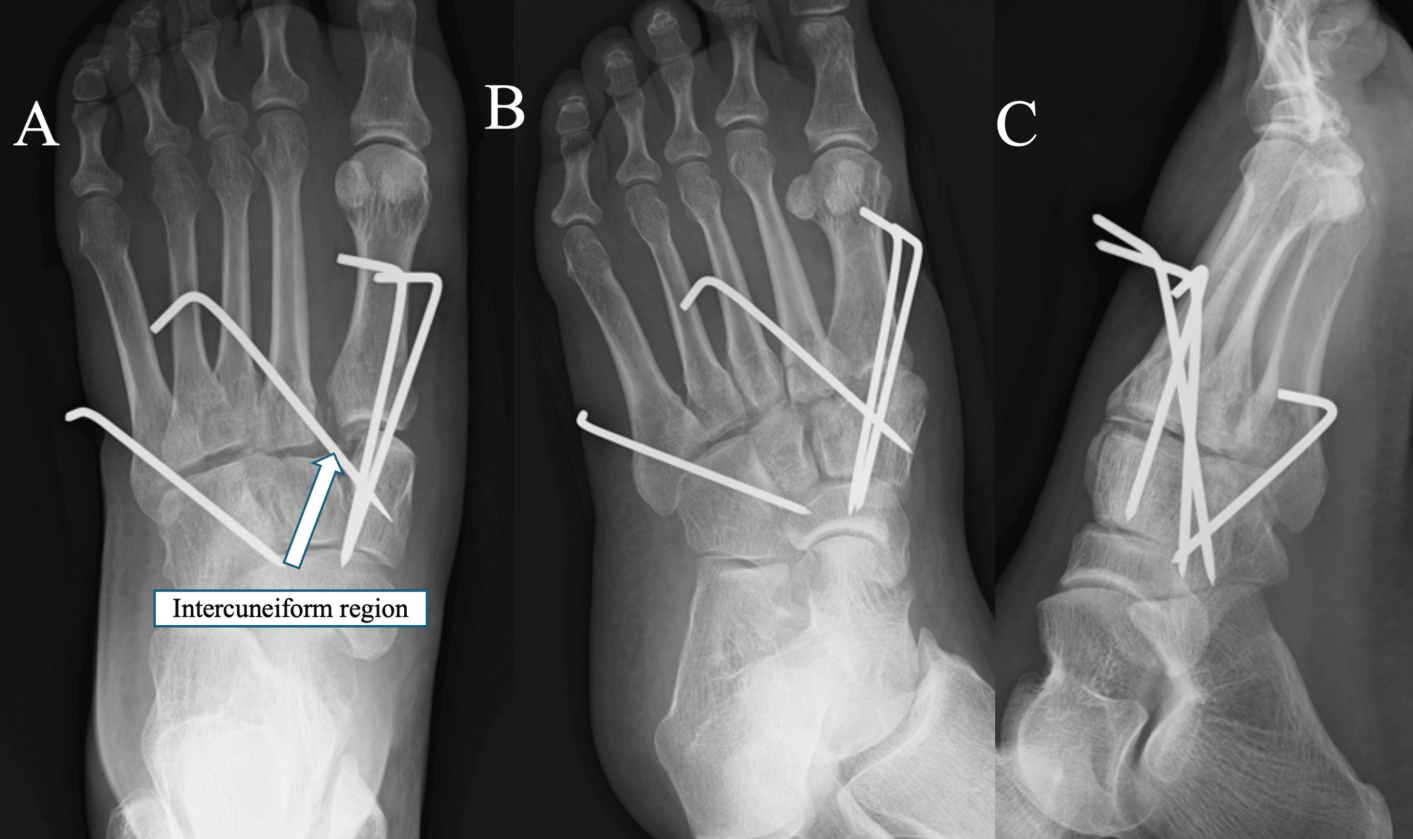
Preoperative radiographs of the left foot after initial emergency surgery (A) Anteroposterior view. (B) Medial oblique view. (C) Lateral view. The images show temporary percutaneous fixation after attempted closed reduction. Arrows and markers indicate the alignment of the medial column and the intercuneiform region, which should be carefully assessed when reduction is incomplete or difficult to maintain.

According to the Myerson classification, this injury was classified as type A, representing total incongruity of the tarsometatarsal joint complex with the metatarsals displaced as a single unit [3]. The patient was transferred to the operating room, where spinal anesthesia was administered. Under C-arm fluoroscopy, closed reduction was attempted. Manual reduction restored alignment only temporarily because congruity at the medial cuneiform-first metatarsal joint was poor, and the dislocation recurred once manual pressure was released. In addition, the widening between the medial and middle cuneiforms persisted after the reduction attempt. These findings suggested the possibility of a mechanical block, including soft-tissue or fracture-fragment interposition. While maintaining acceptable alignment on radiographs, we fixed the fifth metatarsal to the cuboid with a 2.0-mm Kirschner wire (MIZUHO Corporation, Tokyo, Japan). A second Kirschner wire was inserted from the second metatarsal to the medial cuneiform, and the first metatarsal-medial cuneiform joint was stabilized with two wires in total (Figure 2).

**Figure 3:**
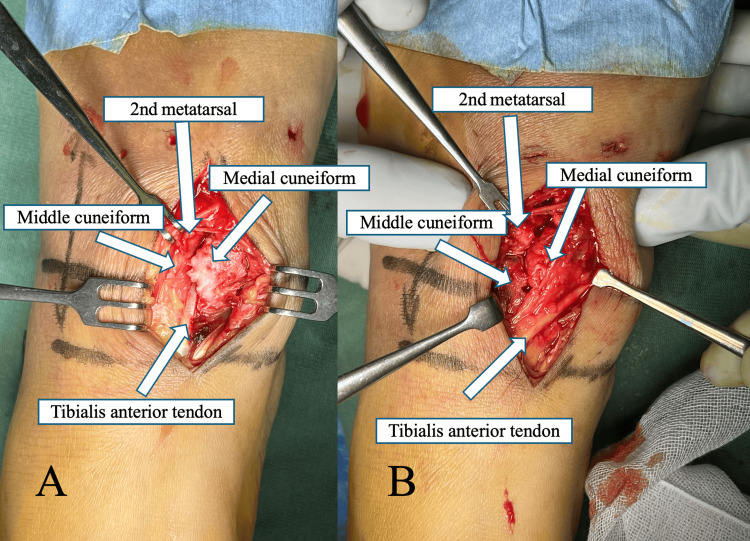
Intraoperative findings (A) The tibialis anterior tendon entrapped between the medial and middle cuneiforms. (B) The tendon after release, with realignment of the joint surfaces and closure of the intercuneiform gap.

Given the marked swelling, only temporary percutaneous fixation was performed on the day of injury, and the patient was immobilized with a below-knee splint and instructed to keep the limb elevated. Definitive internal fixation was planned after the swelling subsided. On postoperative day 14, we performed open reduction and internal fixation. A skin incision was made between the first and second tarsometatarsal joints. The subcutaneous tissues, soft-tissue adhesions, and periosteum were carefully dissected. Intraoperatively, the tibialis anterior tendon was found entrapped between the medial and middle cuneiforms (Figure 3).

**Figure 4:**
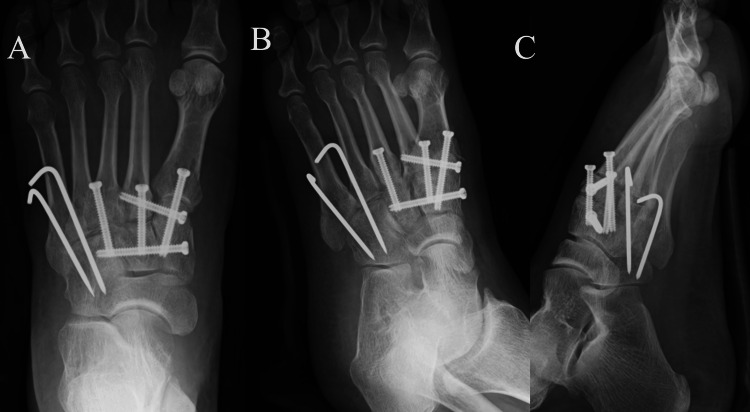
Postoperative radiographs demonstrating definitive fixation (A) Anteroposterior view. (B) Medial oblique view. (C) Lateral view. Cannulated cancellous screws and Kirschner wires (K-wires) were used to stabilize the Lisfranc joint and maintain anatomical alignment of the tarsometatarsal complex.

Once the tendon was released, the gap between the cuneiforms disappeared, and joint alignment was easily restored. The Lisfranc joint was reduced under direct visualization and stabilized. The cuneiform complex was temporarily fixed as a single unit with Kirschner wires, followed by insertion of a 3.5-mm cannulated cancellous screw (MEIRA Corporation, Nagoya, Japan). A second 3.5-mm cannulated cancellous screw was inserted from the medial cuneiform to the second metatarsal to substitute for the ruptured Lisfranc ligament. After adequate compression across the joint surfaces had been confirmed, additional screws were inserted for fixation between the second metatarsal and middle cuneiform, the first metatarsal and medial cuneiform, and the third metatarsal and lateral cuneiform. The fourth and fifth metatarsals were stabilized with 2.0-mm Kirschner wires inserted toward the cuboid (Figure 4).

Postoperatively, a below-knee splint was applied to protect the soft tissues, maintain functional position, and prevent equinus deformity. The splint was maintained for two weeks, after which physical therapy focusing on range-of-motion exercises was initiated. Four weeks after surgery, the Kirschner wires were removed during an outpatient visit. Partial weight bearing was permitted at six weeks, and full weight bearing was initiated at 12 weeks. Bone union was confirmed three months postoperatively. Functional recovery was evaluated at one, three, and six months for dorsiflexion, plantarflexion, inversion, eversion, abduction, adduction, and the American Orthopedic Foot and Ankle Society (AOFAS) midfoot score (Table 1) [12].

**Table 1:** Postoperative foot and ankle ROM and AOFAS midfoot scores at one, three, and six months AOFAS: American Orthopedic Foot and Ankle Society (0-100), ROM: range of motion

Postoperative period	Dorsiflexion (°)	Plantar flexion (°)	Inversion (°)	Eversion (°)	Abduction (°)	Adduction (°)	AOFAS midfoot scores
1 month	10	45	10	10	50	12	82
3 months	20	50	10	8	60	30	87
6 months	25	50	15	10	70	30	90

**Figure 5:**
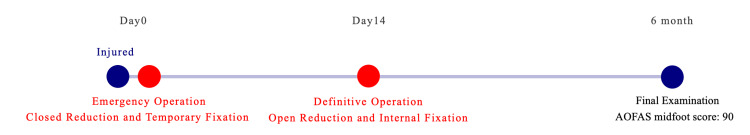
Timeline of the clinical course Timeline from the initial injury and temporary fixation on Day 0 to definitive open reduction and internal fixation on Day 14 and final follow-up at six months. AOFAS: American Orthopedic Foot and Ankle Society (0-100) Image Credit: Authors using PowerPoint (Microsoft Corporation, Redmond, WA, USA)

Table 1 shows progressive improvement in foot and ankle motion over time. At six months postoperatively, the AOFAS midfoot score had improved to 90, indicating favorable short-term functional recovery with minimal pain and favorable performance in daily activities. The overall clinical course is summarized in Figure 5.

**Figure 6:**
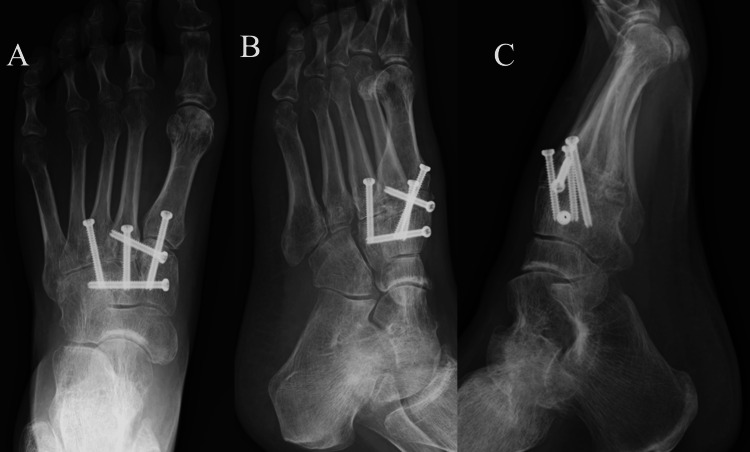
Radiographs of the left foot at six months postoperatively (A) Anteroposterior view. (B) Medial oblique view. (C) Lateral view. Anatomic alignment of the tarsometatarsal joints is maintained, with no evidence of post-traumatic arthritic changes.

Progressive improvements in ankle and foot motion were observed, and at six months postoperatively, the midfoot score had improved to 90. Radiographic evaluation at that time confirmed maintenance of anatomic alignment of the tarsometatarsal joints without signs of post-traumatic arthritis (Figure 6).

## Discussion

Lisfranc fracture-dislocation is a severe injury involving the tarsometatarsal joint complex of the foot. Injury to this region can compromise both mechanical and functional stability and may lead to post-traumatic arthritis or persistent pain even after treatment [1,2,4]. Reported treatment options include percutaneous pinning with Kirschner wires, open reduction and internal fixation with screws or dorsal bridge plates, internal brace augmentation, and primary arthrodesis [5-7,13-18]. However, the optimal procedure, number of operations, and timing of surgery remain controversial.

**Figure 7:**
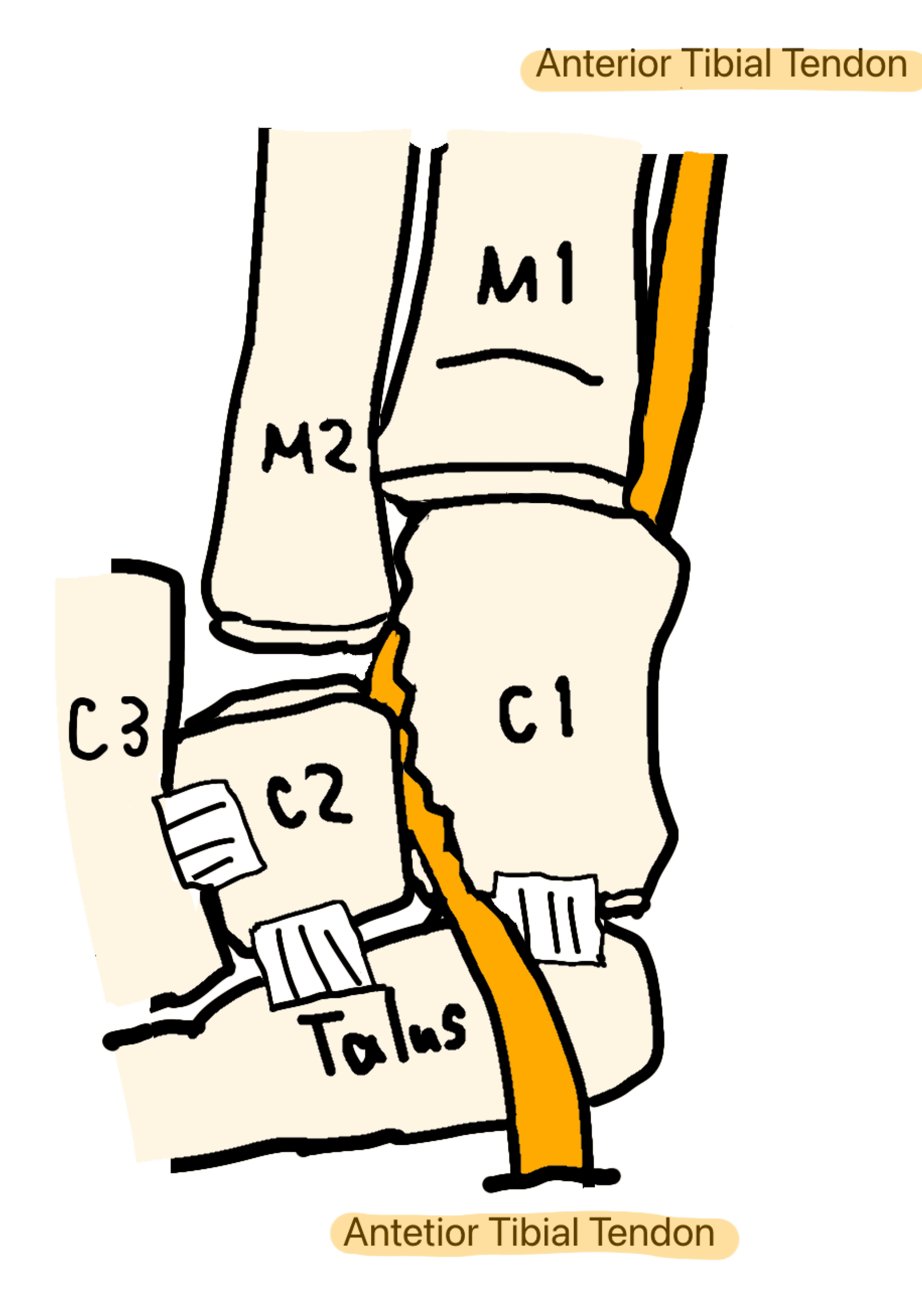
Schematic illustration showing tibialis anterior tendon entrapment between the medial and middle cuneiforms Image Credit: Authors using PowerPoint (Microsoft Corporation, Redmond, WA, USA)

Irreducible Lisfranc dislocations have rarely been reported. Several mechanisms have been described, including tendon entrapment of the tibialis anterior or peroneus longus tendon, incongruity of the medial cuneiform-first metatarsal joint, and interposition of fracture fragments in the second metatarsal-middle cuneiform region [8-11]. In previously reported cases, the tibialis anterior tendon was most commonly entrapped between the medial and middle cuneiforms, and this pattern was often associated with lateral type A Lisfranc dislocations. The present case demonstrated the same pattern, with tendon entrapment between the medial and middle cuneiforms in association with a Myerson type A injury. These similarities suggest that persistent widening between the medial and middle cuneiforms after attempted reduction may be an important clue to tibialis anterior tendon interposition. A schematic illustration of this entrapment mechanism is shown in Figure 7.

The tibialis anterior tendon inserts mainly onto the medial cuneiform and the base of the first metatarsal. Therefore, when the medial column is markedly displaced in a Lisfranc injury, the tendon may be drawn into the space between the medial and middle cuneiforms. In the present case, the Myerson type A lateral dislocation was associated with widening between the cuneiforms, and the tibialis anterior tendon was considered to have become entrapped in this gap. Once the tendon is interposed between the cuneiforms, it can act as a mechanical block, preventing congruent joint reduction and making closed reduction or maintenance of reduction difficult.

In addition to radiographic incongruity, certain clinical findings may help raise suspicion of tendon interposition before surgery. Ashworth et al. described the 'toe-up sign,' in which the great toe remains abnormally extended because of tethering of the tibialis anterior tendon [9]. Persistent deformity, failure to achieve or maintain anatomic reduction despite appropriate manipulation, and widening or incongruity at the medial column should also alert surgeons to the possibility of soft-tissue interposition. Recognition of possible tendon interposition before surgery may help avoid repeated unsuccessful reduction attempts and facilitate timely open reduction. In cases in which anatomic alignment cannot be achieved or maintained, mechanical obstruction should be considered [9-11].

There is an ongoing debate regarding the choice between percutaneous pinning and open reduction with screw fixation for Lisfranc injuries. Mosca et al. suggested that percutaneous pinning can be used to treat Lisfranc fracture-dislocations [15]. However, not all injuries can be managed adequately with pinning, and clear criteria for selecting fracture patterns suitable for this technique have not been established. Previous studies have shown no significant differences in functional outcomes between screw fixation and Kirschner wire fixation when anatomic reduction is achieved, with AOFAS midfoot scores often exceeding 75 points [7,17]. These observations, however, apply only when anatomic reduction can be achieved and may not hold for irreducible injuries, as in the present case. Screw fixation has been reported to provide superior mechanical stability and to facilitate maintenance of anatomic alignment [4,18]. Karaindros et al. reported that lateral type A dislocations accompanied by a gap between the medial and middle cuneiforms are highly suggestive of tibialis anterior tendon interposition and should be managed with open reduction and internal fixation [10]. Similarly, Kim et al. emphasized that when closed reduction and internal fixation fail, surgeons should consider the possibility of tendon or bone-fragment entrapment [11].

In this case, because marked swelling was present on the day of injury, we first performed closed reduction and temporary percutaneous fixation, followed by staged open reduction and internal fixation after the soft-tissue condition improved. The dorsal soft-tissue envelope of the foot is thin, and the extensor tendons run superficially beneath the skin. Extensive surgical exposure in the presence of marked swelling may increase the risk of wound necrosis or soft-tissue defects. Therefore, staged management in this case was selected to balance temporary maintenance of alignment with protection of the soft tissues.

Tibialis anterior tendon interposition was not recognized during the initial pinning procedure and was detected only at second-stage surgery. Despite this delay, the patient achieved favorable short-term outcomes without residual dysfunction of the ankle or toes. At six months postoperatively, the AOFAS midfoot score had improved to 90, and radiographs showed restored alignment of the tarsometatarsal joints without degenerative change. These findings suggest that favorable functional recovery may still be achieved even when tibialis anterior tendon interposition persists for 14 days, provided that the ankle is immobilized, the limb is elevated, and appropriate postoperative rehabilitation is performed.

This case report has several limitations. First, it describes a single patient, and therefore, the findings cannot be generalized to all irreducible Lisfranc injuries. Second, tibialis anterior tendon entrapment was diagnosed intraoperatively and was not confirmed preoperatively by advanced imaging. Third, the follow-up period was limited to six months. Although the patient showed favorable short-term functional recovery and maintained anatomic alignment, longer follow-up is needed to evaluate the durability of the reduction and the development of late complications, including post-traumatic arthritis.

## Conclusions

Tibialis anterior tendon interposition is a rare cause of irreducible Lisfranc fracture-dislocation. When anatomic reduction cannot be achieved or maintained, surgeons should suspect a mechanical obstruction, including tendon entrapment or fracture-fragment interposition. This case suggests that staged surgical management may be a reasonable treatment option in patients with marked soft-tissue swelling, allowing temporary stabilization followed by definitive open reduction and internal fixation once the soft tissues have recovered. However, this favorable outcome is based on a single case with short-term follow-up. Further follow-up is needed to confirm the durability of the clinical and radiographic outcomes and to evaluate late complications, including post-traumatic arthritis.

## References

[1] Moracia-Ochagavía I, Rodríguez-Merchán EC (2019). Lisfranc fracture-dislocations: current management. EFORT Open Rev.

[2] Benirschke SK, Meinberg EG, Anderson SA, Jones CB, Cole PA (2013). Fractures and dislocations of the midfoot: Lisfranc and Chopart injuries. Instr Course Lect.

[3] Myerson M (1989). The diagnosis and treatment of injuries to the Lisfranc joint complex. Orthop Clin North Am.

[4] Philbin T, Rosenberg G, Sferra JJ (2003). Complications of missed or untreated Lisfranc injuries. Foot Ankle Clin.

[5] Myerson MS (1999). The diagnosis and treatment of injury to the tarsometatarsal joint complex. J Bone Joint Surg Br.

[6] Myerson MS, Fisher RT, Burgess AR, Kenzora JE (1986). Fracture dislocations of the tarsometatarsal joints: end results correlated with pathology and treatment. Foot Ankle.

[7] Lau S, Howells N, Millar M, De Villiers D, Joseph S, Oppy A (2016). Plates, screws, or combination? Radiologic outcomes after Lisfranc fracture dislocation. J Foot Ankle Surg.

[8] Denton JR (1980). A complex Lisfranc fracture-dislocation. J Trauma.

[9] Ashworth MJ, Davies MB, Williamson DM (1997). Irreducible Lisfranc injury: the toe-up sign. Injury.

[10] Karaindros K, Arealis G, Papanikolaou A, Mouratidou A, Siakandaris P (2010). Irreducible Lisfranc dislocation due to the interposition of the tibialis anterior tendon: case report and literature review. Foot Ankle Surg.

[11] Kim DY, Kim JK, Kim MW, Lee KB (2021). Irreducible Lisfranc injury by tibialis anterior tendon entrapment: a case report. Medicine (Baltimore).

[12] Kitaoka HB, Alexander IJ, Adelaar RS, Nunley JA, Myerson MS, Sanders M (1994). Clinical rating systems for the ankle-hindfoot, midfoot, hallux, and lesser toes. Foot Ankle Int.

[13] Kirzner N, Zotov P, Goldbloom D, Curry H, Bedi H (2018). Dorsal bridge plating or transarticular screws for Lisfranc fracture dislocations: a retrospective study comparing functional and radiological outcomes. Bone Joint J.

[14] Chen J, Sagoo N, Panchbhavi VK (2021). The Lisfranc injury: a literature review of anatomy, etiology, evaluation, and management. Foot Ankle Spec.

[15] Mosca M, Fuiano M, Censoni D (2021). A mid-term follow-up retrospective evaluation of tarsometatarsal joint fracture-dislocations treated by closed reduction and percutaneous K-wires fixation. Injury.

[16] Rammelt S, Schneiders W, Schikore H, Holch M, Heineck J, Zwipp H (2008). Primary open reduction and fixation compared with delayed corrective arthrodesis in the treatment of tarsometatarsal (Lisfranc) fracture dislocation. J Bone Joint Surg Br.

[17] Stavlas P, Roberts CS, Xypnitos FN, Giannoudis PV (2010). The role of reduction and internal fixation of Lisfranc fracture-dislocations: a systematic review of the literature. Int Orthop.

[18] Lee CA, Birkedal JP, Dickerson EA, Vieta PA Jr, Webb LX, Teasdall RD (2004). Stabilization of Lisfranc joint injuries: a biomechanical study. Foot Ankle Int.

